# Formaldehyde Exposure and Asthma in Children: A Systematic Review

**DOI:** 10.1289/ehp.0901143

**Published:** 2009-11-06

**Authors:** Gerald McGwin, Jeffrey Lienert, John I. Kennedy

**Affiliations:** 1 Department of Epidemiology, School of Public Health, University of Alabama at Birmingham, Birmingham, Alabama, USA; 2 Franklin and Marshall College, Lancaster, Pennsylvania, USA; 3 Department of Veterans Affairs Medical Center, Birmingham, Alabama, USA; 4 Division of Pulmonary, Allergy, and Critical Care Medicine, Department of Medicine, University of Alabama at Birmingham, Birmingham, Alabama, USA

**Keywords:** asthma, children, epidemiology, formaldehyde, meta-analysis

## Abstract

**Objective:**

Despite multiple published studies regarding the association between formaldehyde exposure and childhood asthma, a consistent association has not been identified. Here we report the results of a systematic review of published literature in order to provide a more comprehensive picture of this relationship.

**Data sources:**

After a comprehensive literature search, we identified seven peer-reviewed studies providing quantitative results regarding the association between formaldehyde exposure and asthma in children. Studies were heterogeneous with respect to the definition of asthma (e.g., self-report, physician diagnosis). Most of the studies were cross-sectional.

**Data extraction:**

For each study, an odds ratio (OR) and 95% confidence interval (CI) for asthma were either abstracted from published results or calculated based on the data provided. Characteristics regarding the study design and population were also abstracted.

**Data synthesis:**

We used fixed- and random-effects models to calculate pooled ORs and 95% CIs; measures of heterogeneity were also calculated. A fixed-effects model produced an OR of 1.03 (95% CI, 1.02–1.04), and random effects model produced an OR of 1.17 (95% CI, 1.01–1.36), both reflecting an increase of 10 μg/m^3^ of formaldehyde. Both the *Q* and *I*^2^ statistics indicated a moderate amount of heterogeneity.

**Conclusions:**

Results indicate a significant positive association between formaldehyde exposure and childhood asthma. Given the largely cross-sectional nature of the studies underlying this meta-analysis, further well-designed prospective epidemiologic studies are needed.

Acute exposure to formaldehyde can cause eye, nose, throat, and skin irritation, whereas long-term exposure has been associated with certain cancers (e.g., sinonasal) as well as asthma (Daisey et al. 2003). Exposure to formaldehyde occurs in certain occupational settings (e.g., embalmers), but exposure via formaldehyde-emitting products such as particle board, urea formaldehyde insulation, carpeting, and furniture is more common (Garrett et al. 1999). In the United States, the legal occupational limit for short-term (i.e., < 15 min) formaldehyde exposure is 2 ppm, and the long-term limit (i.e., > 15 min) is 0.75 ppm [Occupational Safety and Health Administration (OSHA) 2005]. In contrast, the National Institute for Occupational Safety and Health suggests that exposure be limited to much lower levels: 0.016 ppm (long term) and 0.1 ppm (short term).

Much of the research regarding the health effects of formaldehyde has focused on cancer, whereas less attention has been paid to more common conditions such as asthma. In the United States, the prevalence of asthma is approximately 7% among adults and 9% among children (Akinbami et al. 2009; Moorman et al. 2007). Among adults, some studies have reported a positive association between formaldehyde and asthma (Wieslander et al. 1997), while others have not (Krzyzanowski et al. 1990). It has been suggested that certain groups, specifically children, may be particularly sensitive to formaldehyde exposure; however, as with adults, the results have been inconsistent, with some studies reporting an association (Garrett et al. 1999) and others not (Symington et al. 1991). All of these studies have specific limitations including small sample sizes (Delfino et al. 2003), the use of self-reported asthma (Smedje et al. 1997), and potential selection bias (Garrett et al. 1999). In addition, the extent of formaldehyde exposure varies widely across studies. For example, Mi et al. (2006) reported a range of 3–20 μg/m^3^, whereas the range reported by Rumchev et al. (2002) was ~ 0–224 μg/m^3^. However, the former study derived measurements from schools while the latter study obtained measurements from homes. Moreover, most studies are cross-sectional and fail to provide information on exposure levels that reflect individual exposure (in magnitude and/or duration).

Although there have been multiple reviews of the literature pertaining to formaldehyde and asthma in children, these have all been qualitative (Mendell 2007). The relationship between formaldehyde and respiratory symptoms has received attention recently because of concerns regarding air quality in mobile homes and travel trailers provided by the Federal Emergency Management Agency (FEMA) to displaced Gulf Coast residents in the aftermath of Hurricane Katrina. We conducted the current study, a systematic review of the literature regarding the potential association between formaldehyde exposure and asthma in children, to shed additional light on the issue.

## Methods

This review was conducted using a modified version of the Meta-analysis of Observational Studies in Epidemiology (MOOSE) guidelines for the conduct of systematic reviews and meta-analysis of observational studies (Stroup et al. 2000). We identified studies through the PubMed/MEDLINE (National Library of Medicine 2009) and Google Scholar (2009) databases, employing a search strategy that combined text word (e.g., “formaldehyde and asthma and children”) and medical subject headings to identify reports regarding formaldehyde exposure and asthma. The reference lists of the identified studies were also reviewed to identify other relevant studies. Studies were initially selected if they appeared to contain qualitative or quantitative estimates for the association between formaldehyde exposure and asthma in children. We were specifically interested in studies that compared children with and without asthma with respect to formaldehyde exposure. All of the studies initially selected were in English.

In total, we identified 18 articles that met the aforementioned criteria, and after a detailed review, determined that 10 articles contained information suitable for use in a systematic review. Three review articles were excluded (Burr 1999; Daisey et al. 2003; Mendell 2007). Three additional articles were excluded because they were not asthma-specific, but rather focused on respiratory symptoms (e.g., chest discomfort) or pulmonary function (Franklin et al. 2000; Symington et al. 1991; Wantke et al. 1996). Two studies were excluded because, although asthma-specific, they did not contain a reference or control group (Delfino et al. 2003; Erdei et al. 2003). For the 10 articles included, we abstracted information regarding study design and setting, subject response/participation rates, definition of asthma (e.g., physician diagnosis), sample size, average (minimum and maximum) formaldehyde levels, average age of study subjects, and quantitative estimates (or raw data) for the association between formaldehyde exposure and asthma as well as whether such estimates were adjusted and, if so, for what measures (Table 1). Three of 10 studies did not contain actual formaldehyde measurements, and attempts to obtain this information from the study authors have been unsuccessful to date (Doi et al. 2003; Pati and Parida 2005; Tavernier et al. 2006).

Once the relevant results from each study were extracted, we determined that homogenizing the individual study results using a single unit of formaldehyde measurement would be necessary. Because most studies reported their results as odds ratios (ORs) per 10-μg/m^3^ unit increase in formaldehyde, this was chosen as the common metric. Thus, results for those studies using different units were transformed. For example, if a study reported an OR reflecting a 1-μg/m^3^ increase in formaldehyde, the natural logarithm of the OR was calculated and multiplied by 10; this value was then exponentiated to obtain an OR for a 10-μg/m^3^ unit increase in formaldehyde. This process was repeated for the 95% confidence interval (CI). Thus, for each study, an OR and 95% CI for the association between asthma and a 10-μg/m^3^ unit increase in formaldehyde exposure was obtained. One study (Zhao et al. 2008) provided two estimates: one for indoor and another for outdoor exposure, both of which were used.

Pooled ORs and 95% CIs were obtained using inverse variance-weighted, fixed-effects, and random-effects models. We tested heterogeneity using the *Q* test and quantified with the *I*^2^ statistic. Whereas the *Q* test only determines whether statistically significant heterogeneity exists, the *I*^2^ statistic calculates the proportion of the variability that can be attributed to heterogeneity across the studies. *I*^2^ values of 25%, 50%, and 75% have been suggested as indicators of low, moderate, and high heterogeneity, respectively. Fixed-effects models are considered appropriate for values of < 50%, whereas for values of ≥ 50%, random-effects models are preferred. To evaluate whether the observed results were unduly influenced by any individual study and to determine if there was any publication bias, an influence plot and a funnel plot, respectively, were used.

## Results

Overall, 10 studies involving 6,387 participants including 635 with diagnosed or self-reported asthma were selected for systematic review, of which seven studies were able to be used in the meta-analysis involving a total of 5,930 participants, 364 of whom had diagnosed asthma (Table 1). Most studies were cross-sectional; half relied on self-reported information on asthma diagnoses, whereas the remainder used actual physician diagnoses. Studies of the former type generally queried participants about whether they had ever been diagnosed with asthma, and thus those responding affirmatively are best characterized as prevalent cases. With respect to those studies using physician diagnosis, based on the study designs as they were described, it was frequently clear that those with asthma would also be best characterized as prevalent cases. In only one study was it entirely clear that the cases were truly incident (i.e., newly diagnosed). Participation rates ranged from 46% to 99%; however, this information was not available for half of the studies.

**Figure 1:**
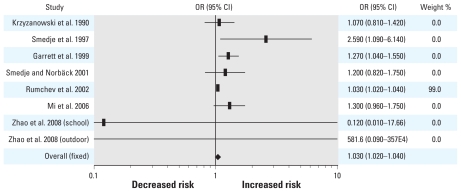
Forest plot of the relative risk estimates and their 95% CIs from the studies included in the meta-analysis of the association between formaldehyde exposure and asthma in children based upon a fixed-effects model.

**Figure 2:**
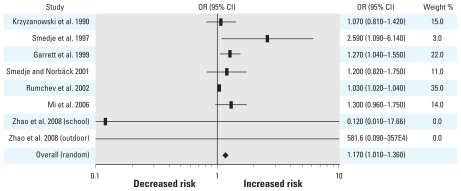
Forest plot of the relative risk estimates and their 95% CIs from the studies included in the meta-analysis of the association between formaldehyde exposure and asthma in children based on a random-effects model.

The results for each individual study as well as the fixed- and random-effects pooled ORs and 95% CIs are shown in Figure 1. The forest plots for both fixed- and random-effects models can be seen in Figures 1 and 2, respectively. The results of the fixed-effects model indicate a 3% increase (95% CI, 1.02–1.04, *p* < 0.0001) in asthma risk for each 10-μg/m^3^ unit increase in formaldehyde, whereas the random-effects model indicates a 17% increase (95% CI, 1.01–1.22, *p* = 0.0158). The *Q* and *I*^2^ statistics were 14.28 (*p* < 0.0001) and 51%, respectively, indicating the presence of moderate between-study heterogeneity.

The influence plot indicated that one study (Rumchev et al. 2002) may have had an undue influence on the study results (data not shown). When this study was excluded, the resulting ORs from fixed- and random-effects models were 1.24 (95% CI, 1.09–1.42) and 1.24 (95% CI, 1.07–1.44), respectively (Table 2). The *Q* and *I*^2^ statistics were 6.76 and 11.2%, respectively.

Table 2 presents the pooled results stratified according to specific study characteristics. Based on the fixed-effects model, the OR for self-reported asthma was 1.22 (95% CI, 1.02–1.46), whereas the OR for diagnosed asthma was 1.03 (95% CI, 1.02–1.04). For the random-effects model, the ORs for self-reported and diagnosed asthma were 1.26 (95% CI, 0.97–1.64) and 1.12 (95% CI, 0.88–1.44), respectively. When stratified according to study design, the ORs for cross-sectional studies were 1.25 (95% CI, 1.08–1.44) for fixed effects and 1.26 (95% CI, 1.03–1.55) for random effects. There was only one cohort study and one case–control study; the results of these individual studies appear in Table 1. With respect to the exposure setting (home vs. school), assuming fixed effects, the OR was 1.03 (95% CI, 1.02–1.04) for home exposure, whereas for school exposure the OR was 1.32 (95% CI, 1.05–1.66). For the random-effects model, the OR for home exposure was 1.10 (95% CI, 0.95–1.27), and the OR for school exposure was 1.33 (95% CI, 1.02–1.74). Only one study (Zhao et al. 2008) evaluated outdoor exposure, and its results appear in Table 1. Finally, the fixed- and random-effects results for the four studies (Mi et al. 2006; Smedje and Norbäck 2001; Smedje et al. 1997; Zhao et al. 2008) that provided participation rates (66%, 82%, 90%, and 99%) were stronger than the results for those for which participation rates were unknown.

The funnel plot did not show evidence of publication bias either with or without Rumchev et al. 2002 (data not shown).

## Discussion

Asthma is a disorder characterized by episodic symptoms and a physiology associated with airway hyperresponsiveness, bronchoconstriction, and excessive mucus production. Fundamentally, asthma is a disorder with inflammation of airways that creates a microenvironment capable of reacting to specific and/or nonspecific stimuli with a stereotypic pathogenic response. Although debate on the topic continues, multiple studies have suggested a link between inhalation exposure to formaldehyde and the development of airway hyperresponsiveness and asthma (Thompson et al. 2008). Several mechanisms have been identified that provide plausible connections between formaldehyde exposure and airways disease. Formaldehyde is a well-recognized irritant affecting multiple tissues; it has been demonstrated to provoke transient decline in pulmonary function (Paustenbach et al. 1997). As a small molecule, formaldehyde may associate with larger protein molecules (e.g., albumin) to create newly antigenic moieties. Such exposure presumably would provoke formation of specific IgE antibodies that could bind to mast cells and, upon subsequent exposure, lead to mast cell degranulation and the elaboration of mediators traditionally associated with the asthmatic (early- and late-phase) response. Alternatively, formaldehyde inhalation, through its nonspecific irritant effect, could provoke mucosal inflammation in airways. If the resultant inflammatory response is T helper-2 dominant, cytokine mediators typically associated with asthma [interleukin (IL)-4, IL-5, IL-9, IL-13] would subsequently be produced (Elias et al. 2003). Recently, formaldehyde has also been demonstrated to alter thiol biology, leading to the accelerated reduction of the endogenous bronchodilator *S*-nitrosoglutathione, thus providing another putative mechanistic link between formaldehyde exposure and airways disease (Thompson et al. 2008).

The results of this study, which pooled the results of seven published studies, suggest a positive relationship between formaldehyde exposure and childhood asthma. To place the observed results (OR of 1.17 per 10-μg/m^3^ increase) in context, when compared with individuals with no formaldehyde exposure, those with the highest levels of exposure reported in the seven studies (i.e., 80 μg/m^3^) would have 3.5-times higher odds of asthma. The results reported herein are consistent with much of the previously published literature regarding the association between formaldehyde exposure and childhood asthma. This should not be surprising in that many of these studies serve as the foundation of our meta-analysis. However, in addition to the seven studies included in the meta-analysis, two additional studies, each reporting a positive association, could not be included, yet they provide further support for the observed quantitative results (Pati and Parida 2005; Tavernier et al. 2006). Tavernier et al. (2006) reported elevated but not statistically significant ORs for specific levels of formaldehyde exposure; unfortunately, the authors did not provide the actual, quantitative values associated with those levels. Similarly, Pati and Parida (2005) simply reported that, “indoor exposure to formaldehyde . . . significantly increased the risk of having asthma.” Additionally, Symington et al. (1991) compared the prevalence of respiratory symptoms exhibited by children living within 1 mile of a formaldehyde-emitting foundry with that of children living in other areas and reported no differences (Symington et al. 1991). Franklin et al. (2000) determined that individuals with a home formaldehyde concentration of at least 50 ppb had a significantly increased volume of exhaled nitric oxide, which serves as a marker of airway inflammation (Franklin et al. 2000). Venn et al. (2003) did not observe an association between persistent wheezing and formaldehyde exposure; however, among children with persistent wheezing, those reporting frequent nighttime symptoms had higher formaldehyde levels compared with those not experiencing nocturnal symptoms (Venn et al. 2003). Erdei et al. (2003) reported a significant increase in immune biomarkers in children exposed to high amounts of formaldehyde (Erdei et al. 2003). Doi et al. (2003) found only 2 of 122 asthmatic children have formaldehyde-specific IgE and concluded that formaldehyde is not a risk factor for childhood asthma (Doi et al. 2003).

By its nature, a systematic review incorporates many individual studies, each of which has its own limitations, and not surprisingly, our analysis revealed low to moderate between-study heterogeneity. Ultimately much of the heterogeneity appears to be attributed to a single study (Rumchev et al. 2002). The reason this study stands out can be partly ascribed to the precision of the OR for the association between formaldehyde and asthma; additionally, this study is unique in that the mean age of the participants was < 2 years. Infants and younger children may be even more vulnerable than older children to the effects of formaldehyde because of the small caliber of their airways. When the analysis was conducted without this study, the results of the fixed- and random-effects models were highly consistent, and there was a decrease in the measures of heterogeneity. Beyond the influence of the Rumchev et al. (2002) study, a number of study-specific limitations must also be mentioned. First, several studies used self-reported information on ever having been diagnosed with asthma (e.g., Krzyzanowski et al. 1990; Smedje and Norbäck 2001). However, research indicates that there is a high level of agreement between self-reported and physician-diagnosed asthma such that this issue is likely of minimal concern (Rönmark et al. 1999). Moreover, when stratified by asthma definition, the results were largely consistent. Another limitation faced by several studies is selection bias (Garrett et al. 1999; Rumchev et al. 2002; Tavernier et al. 2006). For example, Rumchev et al. (2002) hypothesized that because their study focused on indoor environmental risk factors for asthma, it was likely that the people who were most interested in this topic were more likely to participate in the study. The authors also suggested that selection bias may have arisen from low and potentially differential participation rates; however, they describe a number of strategies that were employed to minimize this problem. Although some studies provided adjusted estimates, others did not. Thus, our pooled results may be subject to residual confounding. The extent of this problem is a function of the individual study weights; the two studies that did not provide adjusted estimates generally had higher weights than those that did. Finally, the temporal relationship between etiologically relevant formaldehyde exposure and asthma cannot be established in most of the studies included in our analysis because of their cross-sectional designs. As a result, our pooled results are largely cross-sectional in nature, and the measured formaldehyde levels do not reflect personal exposures. This problem is compounded by the inclusion of those studies wherein “ever asthma” was used as an indication of a positive outcome, because such a definition will capture transient outcomes whose etiology may lie in acute exposures that occurred in the distant past and may not be captured by current exposure levels. The study by Smedje and Norbäck (2001) does not suffer from this potential limitation given its use of a cohort study design. The inability to quantify etiologically relevant formaldehyde levels is attributable to several factors. Formaldehyde levels were not measured at the same time of the year from one study to the next. This is important, because formaldehyde levels can vary with temperature and humidity. Whereas formaldehyde levels were measured at the subjects’ home in some studies, others focused on the school setting. Taken together, the implication is that the observed formaldehyde levels and their association with asthma may not reflect the true the magnitude of formaldehyde exposure, specifically that preceding asthma onset.

## Conclusions

Subject to the limitations discussed above, the results of this systematic review suggest that there is a positive association between formaldehyde levels and childhood asthma. Taken in conjunction with a plausible biological mechanism, the results of this study provide important evidence regarding the potential causal link between formaldehyde and asthma in children. This is not to suggest that closure can be brought to this issue. Well-designed prospective epidemiologic studies are needed to shed additional light on this issue.

## Figures and Tables

**Table 1:** Summary of studies selected for inclusion in meta-analysis.

Source	Setting	Design	Asthma definition	Incident vs. prevalent cases	Participation rate	Exposure	Formaldehyde levels (μg/m^3^)	No. (asthma)	OR (95% CI) per 10-μg/m^3^ increase	Adjusted	Mean age (years)
Krzyzanowski et al. 1990	United States	Cross-sectional	Self-report	Prevalent	Unknown	Home	≤ 50 to > 87.5	298 (47)	1.07 (0.81–1.43)	No	9.3
Smedje et al. 1997	Sweden	Cross-sectional	Self-report	Prevalent	82%	School	< 5 to 10	627 (40)	2.59 (1.10–6.19)	Yes	13–14
Garrett et al. 1999	Australia	Cross-sectional	Diagnosis	Prevalent	Unknown	Home	< 20 to > 50	148 (53)	1.27 (1.04–1.55)	No	10.2
Smedje and Norbäck 2001	Sweden	Cohort	Self-report	Incident	66%	School	< 5 to 72	1,258 (56)	1.20 (0.80–1.70)	Yes	10.3 14.3
Rumchev et al. 2002	Australia	Case–control	Diagnosis	Unclear	Unknown	Home	< 10 to > 60	192 (88)	1.03 (1.02–1.04)	Yes	1.9
Doi et al. 2003	Japan	Case–control	Diagnosis	Prevalent	Unknown	NA	NA	155 (122)	NA	NA	9.4
Mi et al. 2006	China	Cross-sectional	Self-report	Prevalent	99%	School	3 to 20	1,414 (44)	1.30 (0.72–2.32)	Yes	13.0
Tavernier et al. 2006	United Kingdom	Case–control	Diagnosis	Prevalent	46%	Home	NA	130 (65)	a	Yes	8.1
Pati et al. 2005	India	Case–control	Diagnosis	Unclear	Unknown	Home	NA	172 (84)	b	NA	NA
Zhao et al. 2008	China	Cross-sectional	Self-report	Prevalent	90%	School	1 to 5	1,993 (36)	0.12 (0.0008–17.32)	Yes	12.8
Zhao et al. 2008	China	Cross-sectional	Self-report	Prevalent	90%	Outdoor	5 to 7	1,993 (36)	581.59 (0.06–2263796.94)	Yes	12.8

NA, not applicable.

a Elevated (nonsignificant) ORs for living room and bedroom formaldehyde levels.

b Indoor exposure to formaldehyde significantly increased the risk of asthma.

**Table 2:** Pooled ORs and 95% CIs for fixed- and random-effects models.

		Fixed effects				Random effects	
	No. of studies	OR (95% CI) per 10-μg/m^3^ increase	*p*-Value	*Q*	*I*^2^	OR (95% CI) per 10-μg/m^3^ increase	*p*-Value
All studies	7	1.03 (1.02–1.04)	< 0.0001	14.28	51.0	1.17 (1.01–1.36)	0.0202
Excluding Rumchev et al.	6	1.24 (1.09–1.42)	0.0013	6.76	11.3	1.24 (1.07–1.45)	0.0026
Diagnosis method							
Self-reported	6	1.21 (1.02–1.46)	0.0158	6.66	24.9	1.26 (0.97–1.64)	0.0446
Diagnosed	2	1.03 (1.02–1.04)	< 0.0001	4.22	76.3	1.12 (0.88–1.44)	0.1711
Study design							
Cohort	1	1.20 (0.80–1.70)	0.1711				
Case–control	1	1.03 (1.02–1.04)	< 0.0001				
Cross-sectional	6	1.25 (1.08–1.44)	0.0013	6.72	25.6	1.26 (1.03–1.55)	0.0122
Exposure setting							
Home	3	1.03 (1.02–1.04)	< 0.0001	4.29	53.4	1.10 (0.95–1.27)	0.1056
School	4	1.32 (1.05–1.66)	0.0082	3.48	13.8	1.33 (1.02–1.74)	0.0179
Participation rate							
> 60%	4	1.34 (1.00–1.81)	0.0519	5.66	29.3	1.43 (0.92–2.23)	0.1139
Unknown	3	1.03 (1.02–1.04)	< 0.0001	4.29	30.1	1.09 (0.96–1.25)	0.1924
